# Run-in periods and treatment outcomes in asthma trials: A narrative review

**DOI:** 10.1016/j.conctc.2024.101382

**Published:** 2024-10-15

**Authors:** Emilio Pizzichini, Guy Brusselle, Dawn Edwards, Peter G. Gibson, Huib A. Kerstjens, Alison Moore, David Slade, Robert A. Wise, Shiyuan Zhang

**Affiliations:** a GSK, London, UK; b Federal University of Santa Catarina, Santa Catarina, Brazil; c Ghent University Hospital, Ghent, Belgium; d Department of Respiratory and Sleep Medicine, John Hunter Hospital, Hunter Medical Research Institute, Newcastle 2305, Australia; e Centre of Excellence in Severe Asthma, University of Newcastle, Newcastle 2308, Australia; f University of Groningen and University Medical Center Groningen and Groningen Research Institute for Asthma and COPD, Groningen, the Netherlands; g GSK, Research Triangle Park, NC, USA; h Johns Hopkins University School of Medicine, Baltimore, MD, USA; i GSK, Collegeville, PA, USA

**Keywords:** Asthma therapy, Asthma outcomes research, Asthma exacerbations

## Abstract

**Background:**

The run-in period is an important element of randomized controlled trials, and is often used in respiratory disease trials. The design of the run-in period can greatly impact results and data interpretation, and as such should be designed carefully.

**Methods:**

In this review, we describe the design of run-in periods across six phase 3A trials of triple therapy in asthma, and discuss how differences in run-in period design (specifically the duration, treatment, and reporting of run-in results) may have the potential to alter the interpretation of study outcomes.

**Results:**

We found that the duration of run-in periods ranged between 2 and 7 weeks, with some studies including a combination of screening, run-in and stabilization periods, and others including a run-in period only. Run-in treatment also varied, with some studies running in patients on their previous inhaled corticosteroid/long-acting β_2_-agonist (ICS/LABA) therapy, and others harmonizing treatment by switching to the same ICS/LABA combination used in the on-treatment phase, or a different ICS/LABA combination entirely. Most of the studies included did not report any changes to study outcomes seen prior to randomization.

**Conclusion:**

We discuss the potential implications associated with the various trial designs, and propose that run-in periods should be consciously designed to meet the goals of the specific study. We also propose that standardized reporting of run-in changes would further allow for differentiation between improvements due to improved adherence and true treatment benefits, and aid with comparing data from different clinical trials.

## Highlights

• Variation exists in run-in periods design among phase 3A asthma triple therapy trials.
• Run-in periods to be consciously designed with goals of the specific trial in mind.
• Run-in periods standardized reporting would aid comparing data from clinical trials.

## 1 Background

Randomized controlled trials (RCTs) are critically important to assess the effects of a particular treatment on a defined population of patients [1], as part of the regulatory approval process of new medicines, and to form the evidentiary basis for recommendations made in respiratory clinical practice guidelines [2,3]. The design and conduct of an RCT is of the utmost importance, underpinning the validity of study outcomes [4]. One element of RCTs often used in respiratory disease trials is the run-in period – the period between the screening visit and randomization to study treatments – the design of which requires close consideration [5].

Some potential goals of a run-in period [6] are summarized in Table 1, and include: ensuring that the potential effects of improved adherence from participating in an RCT are fully achieved before randomization to study treatments; stabilizing the patient population's disease (whether on the same or different treatment); and harmonizing background treatment (i.e. same compound in a class and same delivery device).

**Table 1:** Potential goals of a run-in period.

Confirmation that patients meet the trial enrolment criteria

Familiarization of patients with key outcome measures (e.g. spirometry)

Familiarization of patients with new devices/inhalers

Testing of methodologies (e.g. diaries, spirometers, inhalation techniques)

Ensuring that the potential effects of improved adherence from participating in an RCT are fully achieved before randomization to study treatments

Stabilizing the patient population's disease (whether on the same or different treatment)

Harmonization of background treatment (i.e. same compound in a class and same delivery device)

Obtaining robust baseline measurements

Ensuring medication compliance

Washing out the effects of prior respiratory therapies

Reducing the impact of regression to the mean by distinguishing the measures of eligibility at screening from the baseline measures used to calculate post-randomization changes

Removal of patients for whom changing therapy may be unsafe

RCT, randomized controlled trial.

Run-in periods should be designed in accordance with the objectives of the study and would therefore vary depending on the clinical phase. For example, with the distinct primary objective of an early-stage pharmacokinetic study compared with a large phase 3A registration study, different run-in period designs would be necessary in order to optimize each study's design for the objectives being evaluated.

For a particular class of therapy and patient population, we would expect the design of registrational phase 3A studies to be sufficiently similar so as to enable assessment of relative clinical effects. Any major differences should be critically assessed to identify potential clinical considerations and best practices for future studies. There have been a number of phase 3A triple therapy inhaled corticosteroid/long-acting β_2_-agonist/long-acting muscarinic antagonist (ICS/LABA/LAMA) asthma studies published within the past few years, resulting in the approval of several triple therapy options for patients with asthma who are uncontrolled on ICS/LABA therapies. In this review, we describe the design of run-in periods across these phase 3A triple therapy in asthma trials (with no time-restriction) and discuss how differences in run-in period design may have the potential to alter the interpretation of study outcomes such as lung function, exacerbations and patient-reported outcomes.

Several considerations regarding run-in period design and implications for study outcomes will be discussed throughout the article: 1) longer run-in periods may be associated with improved treatment adherence; 2) detection of run-in improvements in disease characteristics during the run-in period may distinguish study intervention effects from those due to clinical trial participation (e.g. adherence, device familiarity, Hawthorne effect), thus improving the internal validity of the study; similarly, run-in improvements may disqualify patients from randomization; 3) the study population and outcomes of a study may be impacted by whether the run-in treatment is a continuation of patients’ therapy prior to the study, or whether patients receive a treatment type and/or dose different from their previous therapy during run-in.

## 2 Methods

### 2.1 Search strategy

A literature search was performed in March 2023 using PubMed (National Institutes of Health, Bethesda, Maryland, USA) to identify phase 3A RCTs of at least 24 weeks’ duration, examining inhaled triple therapies (ICS/LABA/LAMA) versus the same ICS/LABA for asthma with no date restrictions.

### 2.2 Identified studies

We identified a total of four manuscripts reporting primary results from six phase 3A studies in triple therapy for asthma (summarized in Table 2).

**Table 2:** Asthma study design.

Study	Study type	Patient population	Post-randomization treatment comparison	Run-in period			Post-randomization outcomes		
				Duration/treatment	Criteria checks	Measures of asthma control	Pre-dose FEV_1_ change from baseline^a^	Exacerbations^b^	ACQ-7 change from baseline^c^
CAPTAIN [8]	**Phase 3A,** randomized, double-blind, 24–52-week, active-controlled, parallel-group study	Uncontrolled asthma (ACQ-6 ≥1.5) despite maintenance ICS/LABA therapy for ≥12 weeks Healthcare contact or temporary change in asthma therapy in the year prior to screening (no requirement for history of exacerbations)	FF/UMEC/VI (100/62.5/25 μg) QD vs FF/VI (100/25 μg) QD	Pre-screening: 0–2 weeks Run-in: 3 weeks open-labelFP/SAL 250/50 μg BID Stabilization: 2 weeks open-label FF/VI 100/25 μg QD	Eligibility criteria (at screening) Enrolment criteria (following run-in) Randomization criteria (following stabilization)	ACQ-6 (screening and following run-in)	110^d^ (66, 153) p < 0.0001	0.78 (0.61, 1.01) p = 0.06	−0.116^d^ (−0.210, −0.023) p = 0.015
			FF/UMEC/VI (200/62.5/25 μg) QD vs FF/VI (200/25 μg) QD				92^d^ (49, 135) p < 0.0001	0.97 (0.73, 1.28) p = 0.80	−0.062^d^ (−0.156, 0.032) p = 0.20
IRIDIUM [9]	**Phase 3,** randomized, double-blind, 52-week, active-controlled, parallel-group study	Uncontrolled asthma (ACQ-7 ≥1.5) despite maintenance ICS/LABA therapy for ≥3 months History of ≥1 asthma exacerbation in the year prior to screening	MF/GLY/IND (80/50/150 μg) QD vs MF/IND (160/150) QD	Screening: 2 weeks medium- to high-dose ICS/LABA Run-in: 2 weeks medium-dose open-label FP/SAL 250/50 μg BID	Eligibility criteria (at screening) Enrolment criteria (prior to run-in) Randomization criteria (at randomization)	ACQ-7 (prior to run-in and at randomization)	76^e^ (41, 111) p < 0.001	0.87 (0.71, 1.06) p = 0.17	−0.071^e^ (−0.151, 0.010) p = 0.085
			MF/GLY/IND (80/50/150 μg) QD vs FP/SAL (500/50) BID				99^e^ (64, 133) p < 0.001	0.81 (0.66, 0.99) p = 0.041	−0.084^e^ (−0.164, −0.005) p = 0.038
			MF/GLY/IND (160/50/150 μg) QD vs MF/IND (320/150) QD				65^e^ (31, 99) p < 0.001	0.85 (0.68, 1·04) p = 0·12	0.014^e^ (−0.660, 0.094) p = 0.73
			MF/GLY/IND (160/50/150 μg) QD vs FP/SAL (500/50) BID				119^e^ (85, 154) p < 0.001	0.64 (0.52, 0.78) p < 0·001	−0.086^e^ (−0.165, −0.006) p = 0.034
TRIGGER [7]	**Phase 3**, randomized, double-blind, 52-week, active-controlled, parallel-group study	Uncontrolled asthma (ACQ-7 ≥1.5) despite stable, high-dose maintenance ICS/LABA therapy for ≥4 weeks History of ≥1 asthma exacerbation in the year prior to screening	BDP/GLY/FOR (200/6/10 μg) BID vs BDP/FOR (200/6 μg) BID	Run-in: 2 weeks open-label BDP/FOR (200/6 μg) BID	Eligibility criteria (at screening) Randomization criteria (at randomization)	ACQ-7 (at screening and at randomization)	73^e^ (26, 120) p = 0.0025	0.88 (0.75, 1.03) p = 0.11	NR
TRIMARAN [7]	**Phase 3,** randomized, double-blind, 52-week, active-controlled, parallel-group study	Uncontrolled asthma (ACQ-7 ≥1.5) despite stable, medium-dose maintenance ICS/LABA therapy for ≥4 weeks History of ≥1 asthma exacerbation in the year prior to screening	BDP/GLY/FOR (100/6/10 μg) BID vs BDP/FOR (100/6 μg) BID	Run-in: 2 weeks open-label BDP/FOR (100/6 μg) BID	Eligibility criteria (at screening) Randomization criteria (at randomization)	ACQ-7 (at screening and at randomization)	57^e^ (15, 99) p = 0.0080	0.85 (0.73, 0.99) p = 0.033	NR
PrimoTinA-1 [10]	**Phase 3,** randomized, double-blind, 48-week, placebo-controlled, parallel-group study	Uncontrolled asthma (ACQ-7 ≥1.5) despite maintenance ICS/LABA therapy for ≥4 weeks History of ≥1 asthma exacerbation in the year prior to screening	Regular maintenance therapy with add on TIO 5 μg vs placebo QD	Screening: 4 weeks continue with existing ICS (≥800 μg BUD or equivalent dose of another ICS) and a LABA	Eligibility criteria (at screening)	ACQ-7 (at screening)	88^d^ (27, 149) p < 0.01	Endpoint not comparable: time-to-first severe exacerbation available	−0.13^d^ (−0.26, 0.01) p = NS
PrimoTinA-2 [10]	**Phase 3,** randomized, double-blind, 48-week, placebo-controlled, parallel-group study	Uncontrolled asthma (ACQ-7 ≥1.5) despite maintenance ICS/LABA therapy for ≥4 weeks History of ≥1 asthma exacerbation in the year prior to screening	Add on TIO 5 μg vs placebo QD	Screening: 4 weeks continue with existing ICS (≥800 μg BUD or equivalent dose of another ICS) and a LABA	Eligibility criteria (at screening)	ACQ-7 (at screening)	111^d^ (53, 169) p < 0.001	Endpoint not comparable: time-to-first severe exacerbation available	−0.2^d^ (−0.3, −0.07) p < 0.01

ACQ, Asthma Control Questionnaire; BDP, beclomethasone dipropionate; BID, twice daily; BUD, budesonide; FEV_1_, forced expiratory volume in 1 s; FF, fluticasone furoate; FOR, formoterol fumarate; FP, fluticasone propionate; GLY, glycopyrronium bromide; ICS, inhaled corticosteroid; IND, indacaterol; LABA, long-acting β_2_-agonist; MF, mometasone furoate; NR, not reported; NS, not significant; QD, once daily; SAL, salmeterol; TIO, tiotropium; UMEC, umeclidinium; VI, vilanterol.

a Change from baseline pre-dose FEV_1_ (mL, mean difference [95 % CI]).

b Annualized rate moderate/severe exacerbations (rate ratio [95 % CI]), unless specified otherwise.

c Points difference [95 % CI].

d At week 24.

e At week 26.

Although all of the studies identified included some form of run-in period, the terminology used to describe the periods varied (Table 2). In general, a “screening period” comprised the period between screening and randomization during which patients continued on their usual asthma treatment, whereas during a “run-in” period, patients generally received a study-specific run-in treatment. A “pre-screening” period was seen in one study, which was similar to a screening period, except it occurred prior to the screening visit. One study had a run-in period on a run-in ICS/LABA treatment, followed by a “stabilization period” on the ICS/LABA that was used post-randomization. Here, we use the phrase “run-in period” to cover run-in periods, screening periods and stabilization periods for simplicity, and specify further when needed.

## 3 Results

### 3.1 Run-in period duration

**Fig. 1:**
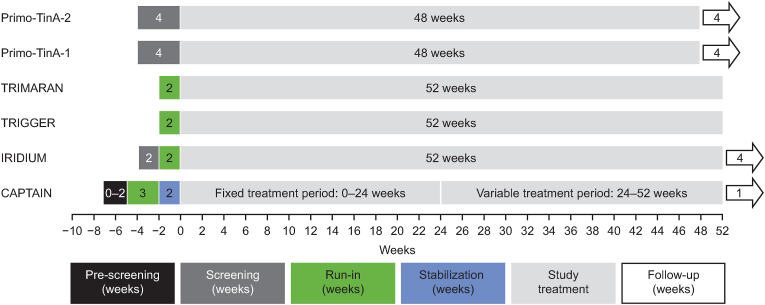
Study periods.

There was considerable variation in the duration of the run-in periods, which ranged between 2 and 7 weeks when including screening periods, and between 0 and 5 weeks when not including screening periods (Table 2 and Fig. 1).

The TRIGGER and TRIMARAN studies [7] had the shortest run-in periods, each with a 2-week run-in. In contrast, the CAPTAIN trial [8] had the longest run-in period (5 weeks) comprising a 3-week ‘run-in’ and a 2-week “stabilization” period; it was the only study to include separate run-in and stabilization periods. In CAPTAIN, patients were “run-in” on a standard ICS/LABA (medium-dose fluticasone propionate/salmeterol) to “wash out” their regular ICS/LABA maintenance therapy on which they entered the study, and then stabilized with a dual therapy (fluticasone furoate/vilanterol [FF/VI] 100/25 μg). The dual therapy used during stabilization was the same inhaler used post-randomization in order to familiarize patients with the new inhaler and achieve optimum adherence.

Including both a run-in and a stabilization period in the CAPTAIN study, with a requirement for uncontrolled asthma following the 3-week run-in phase, allowed for the exclusion of patients who became controlled following adherence to the run-in treatment (fluticasone propionate/salmeterol). As such, patients whose asthma remained uncontrolled despite ICS/LABA were included in the trial, and not simply those who would become controlled when correctly adherent. A robust run-in/stabilization period such as the one seen in CAPTAIN also contributes to ensuring that any post-randomization changes are true effects of the study drug, as opposed to a result of improved adherence. Consequently, longer and more robust run-in periods may reveal fewer post-randomization changes when comparing with studies with shorter run-in periods, as a result of clinically important improvements that may have arisen prior to randomization that would not have been discovered in a study with a shorter run-in period. Therefore, it may be difficult to compare data between trials with substantially differing run-in periods, particularly when considered in addition to other inter-trial differences, such as varying inclusion criteria.

Long run-in periods may identify patients who are more likely to adhere to and correctly use medications (and potentially exclude those who are not), which could improve the efficiency and statistical power of the trial [5]. To assess this, the proportion of patients excluded prior to randomization was collated for each study, along with compliance as measured in the pre- and post-randomization periods. However, we identified a clear data gap with none of the included studies reporting run-in adherence, and only CAPTAIN reporting adherence during the study period [[8], [9], [7], [10]].

There are also pharmacologic/pharmacodynamic effects to consider when deciding upon the duration of a run-in period. The time for maximal effect of ICS to be achieved has been reported to be 3–6 months for some outcomes, and 4–8 weeks for LAMAs [[11], [12], [13], [14], [15]]. As such, for many of the studies included in Table 2, maximal effect for the ICS components of the run-in treatment is unlikely to have been reached prior to randomization. Given it may be impractical to implement a 3- to 6-month run-in period for most studies, ensuring minimal variability when patients achieve the maximal ICS effect is crucial. Indeed, study inclusion criteria will assist in minimizing such variation; however, the run-in period may also play a role. Whether some patients enrolled are ICS-naïve or were previously taking a different ICS compound, this may differentially impact when the maximal ICS effect is reached. Thus, run-in periods where patients all receive the same therapy, as implemented in CAPTAIN, TRIMARAN, TRIGGER, and IRIDIUM, may assist in minimizing the effect of previous treatments. The additional wash-out period included in CAPTAIN may further reduce the effect of prior medications on the time to maximal ICS effects. Given all the identified studies have primary endpoints at approximately 6 months, some patients may only begin to experience maximal effects toward the end of the study. Thus, the benefits of long-term exposure to maximal ICS effects may be observed only in the subsequent months. Therefore, assessing stochastic events such as exacerbations, beyond 6 months may lead to more robust effect sizes in the absence of a sufficiently long run-in period. This would not be the case for lung function or patient-reported outcomes, as they would be expected to manifest before 6 months.

Despite the benefits of a long, robust run-in period already discussed, there are some potential negative aspects to consider when deciding on the most appropriate run-in duration. For example, longer run-in periods may be less reflective of real-world clinical practice. In addition, longer run-in periods may result in increased trial administration costs, greater inconvenience to the patient (e.g. increased number of visits, increased numbers of forms/questionnaires to complete), and more patient withdrawals for instance due to exacerbations, leading to selection bias. Additionally, there may be a greater number of randomization failures due to additional adherence requirements during the run-in period, resulting in a longer study with larger number of patients needed for screening/run-in.

Finally, it may be argued that the post-randomization comparison arm, which is also designed to address issues around adherence/stabilization on treatment/regression to mean and is longer than the run-in periods, negates the requirement for a longer run-in. This is particularly pertinent given that in all studies examined here there was a positive effect of adding a LAMA on forced expiratory volume in 1 s (FEV_1_), as such a longer run-in may not be needed.

Given that the main goals of a phase 3A study are to generate statistically significant and clinically relevant evidence of benefits of a new treatment versus standard, then longer run-in periods are valuable in determining the true treatment impact. Shorter run-in periods, which may be more representative of real-world clinical practice, may be better suited to phase 3B–4 studies designed to reflect real-world results. However, benefits of a longer run-in period must be carefully weighed up with the specific goals of the study, and potential downsides of increasing the run-in length discussed above.

### 3.2 Run-in period treatments

As with the duration of the run-in period, the treatment(s) received during the run-in period should be carefully selected to ensure the aims of the study are met.

Similar to run-in length, substantial variation was observed among the identified studies. While the single-inhaler triple therapy studies (CAPTAIN [8], IRIDIUM [9], TRIGGER and TRIMARAN [7]), all switched patients from their previous therapy to a standard ICS/LABA therapy for the run-in period; the add-on tiotropium studies (PrimoTinA-1/2 [10,16]) had patients continue on their regular asthma medication (and the intervention phase; i.e. screening period only). The decision as to whether to keep patients on their previous ICS/LABA therapy or to switch all patients to a specified run-in therapy should be carefully considered based on study goals. While patients continuing on their regular asthma medication reflects real-world practice, and thus may improve the external validity of the study, the fact that the study population is not harmonized on the same medication makes the effects of the study treatment more difficult to accurately measure (i.e. reduced internal validity). Ensuring all patients are harmonized on dual ICS/LABA therapy may also help to enrich patients who need additional therapy rather than those who become controlled when fully adherent to ICS/LABA. Logistical considerations may also impact this decision (i.e. allowing patients to remain on their regular therapy may improve study recruitment).

IRIDIUM differs slightly from the other single-inhaler triple therapy studies in that it did not run-in patients on the same ICS/LABA combination used in the on-treatment phase (unlike CAPTAIN, TRIGGER and TRIMARAN); thus, there is the potential that patients in IRIDIUM were not as well stabilized as those in other studies. In the CAPTAIN study, as patients received FF/VI 100/25 μg during the stabilization phase, both the effects of adding UMEC and/or the effects of increasing the dose of FF could be studied. One potential issue with the harmonized treatment of all patients on the same ICS/LABA treatment in CAPTAIN is that for patients randomized to the FF/VI 200/25 μg reference arm, there was a step-up in medication at baseline. This may alter interpretation, particularly in comparison to those patients who were randomized to the FF/VI 100/25 μg reference arm (i.e. the same as the stabilization phase treatment). One potential option for clinical trials such as CAPTAIN where multiple ICS/LABA doses are tested could be to run-in patients who were previously on high-dose ICS/LABA on the higher dose of the study drug, and then randomize them to the higher ICS dose-containing triple therapy.

Finally, behavioral/psychological factors must be accounted for, recognizing the potential for patients accessing healthcare more frequently due to a forced change in medication during the run-in period, as seen during the Salford lung study [17,18]. This could ultimately have a negative impact on disease control among patients forced to switch medication.

In the context of a phase 3A trial, in which the aim is to demonstrate the true effect of the new medicine versus a standard of care, our view is that whether to run-in patients on a harmonized therapy should be chosen with utmost care, as they may crucially impact the outcomes. It should be carefully considered whether the benefits of having all patients harmonized on the same run-in therapy outweighs the benefits of having a population that is more reflective of the “real-world” within the context of the particular study, and behavioral/psychological factors should be factored in.

### 3.3 Reporting of run-in results

As noted previously, entering a patient into a long run-in period may result in clinically important improvements prior to randomization, influencing the clinical outcomes of the study in the post-randomization phase. This is a particularly important consideration for phase 3A registration studies, in which the goal is to establish the safety and efficacy of a new drug. Among the included studies, only CAPTAIN [8] reported changes in lung function and asthma control observed during the run-in period*.* Across the 5-week run-in/stabilization period in CAPTAIN, substantial mean improvements were seen in pre-bronchodilator FEV_1_ (287 mL increase), ACQ-7 score (0.67 decrease) and ACQ-6 score (0.64 decrease). As such, lung function and asthma control had improved at randomization relative to screening, thereby reducing the potential for further improvement following randomization to study treatment.

While the baseline characteristics at randomization are generally reported in study publications, accurate and comprehensive reporting of any run-in changes in study outcomes rarely are. Reporting of run-in changes would aid in interpreting results as it would provide additional context so that readers may have a better understanding of the improvements that took place prior randomization (likely due to improvement in adherence, inhaler technique, etc.) and how this impacts the post-randomization results when compared to other studies.

## 4 Discussion

Having reviewed the designs of the studies included in this review, we believe there are several important considerations that would help to improve the ease of comparison with other study results as well as the clinical relevance of study results for patients with asthma in the future.

### 4.1 Study design

The duration and treatment used during the entire run-in phase of a study should be given careful consideration, with run-in periods deliberately designed to address the goals of the particular study. The optimal run-in period duration/treatment is dependent on specific study goals, and the pros and cons of longer run-in periods on harmonized therapy versus shorter screening periods on regular therapy should be balanced. Of note, elements such as re-education, support, adherence, and inhaler technique are crucially important in delivering the full benefit of a new respiratory therapy, and should be addressed during the run-in stage to ensure the true clinical benefit of a drug can be demonstrated. In particular, it is the authors’ belief that generally, adherence should always include adherence with study procedures and visit, not just with study drug administration. Arguably, data loss is a greater threat to study validity in phase 3 than failure of adherence to study drug.

Finally, the time-to-maximal response of the study drugs should be taken into account when deciding on study period length, though this will need to be balanced with the realities of designing and carrying out clinical trials.

### 4.2 Study reporting

Reporting of pre-randomization changes in all clinical trials would allow differentiation between true treatment response and improvements seen due to adherence, and allow for easier comparisons between similar trials. The Consolidated Standards of Reporting Trials (CONSORT) Statement currently only recommends the reporting of baseline demographics and characteristics [1], the authors are of the opinion that reporting of pre-randomization changes should become part of the CONSORT recommendations for the optimal reporting of RCTs. In order to understand the impact of run-in periods on study outcomes, we recommend future asthma trials should report on the following pre-randomization data: changes in symptom control, changes in lung function, adverse events, inhaler technique, treatment adherence.

### 4.3 Interpretation and the potential for under- or over-estimation of clinical effects in practice

Differences between study populations and study methodologies make head-to-head comparisons between RCTs challenging. In order to further understand the impact of different run-in factors (such as duration, treatment medication and inclusion/exclusion criteria following run-in) on outcome variables (e.g. change from screening to randomization in FEV_1_, symptoms), modelling approaches using data from individual studies may be of value. Notably, though the application of robust run-in periods may aid in accurately reporting true impacts of the study drug, they could reduce the generalizability of results from clinical trials to real-world populations.

Future studies could include a treatment arm in which patients receive the study drug of interest during the run-in period in order to generate data that are relevant to real-world clinical practice by showing actual changes that can be expected after a direct switch to a new treatment directly from a previous therapy.

### 4.4 Treatable traits

Given the recent move towards personalized asthma therapy [19], and evidence that biomarkers of type 2 inflammation (namely blood eosinophils and fractional exhaled nitric oxide [FeNO]) may predict inhaled treatment outcomes [8], an additional consideration for future trials is measurement of blood eosinophil levels and FeNO during run-in. This may help determine whether asthma phenotype may impact on the outcomes of the study treatment.

## 5 Conclusion

Substantial variation exists in the run-in periods implemented in recent asthma triple therapy phase 3A clinical trials. In the CAPTAIN trial, a long and robust run-in and screening period, including 2 weeks of treatment on the same ICS/LABA therapy used in one of the comparator arms, resulted in clinically significant and meaningful improvements in outcomes before the treatment under investigation had been initiated. This suggests that in studies with shorter/less robust run-in periods, some of the improvement versus baseline attributed to the investigational product may actually be due to study effects, which should become visible as a similar effect in the placebo arm. Conversely, longer run-in periods may be less reflective of real life, may increase clinical trial costs and inconvenience to patients, and may not be necessary if the post-randomization comparator arm sufficiently addresses issues surrounding adherence/stabilization on treatment/regression to mean. The pros and cons must be carefully weighed in the context of each individual study. In addition, selection of run-in treatments and the length of the run-in period of clinical trials must be taken into account when interpreting clinical study results. Future respiratory trials may benefit from longer run-in and/or stabilization periods to allow run-in treatments the time to take effect, and to ensure improved adherence and inhaler technique during the study treatment period does not impact results. Standardized reporting of pre-randomization changes would further allow for differentiation between improvements due to improved adherence and true treatment benefits, and aid with comparing data from different clinical trials.

In summary, run-in periods are an important aspect of clinical trials necessitating conscious design in the context of the specific aim of the trial, and due consideration when interpreting trial data.

## CRediT authorship contribution statement

**Emilio Pizzichini:** Writing – review & editing, Writing – original draft, Data curation. **Guy Brusselle:** Writing – review & editing, Writing – original draft, Data curation. **Dawn Edwards:** Writing – review & editing, Writing – original draft, Data curation. **Peter G. Gibson:** Writing – review & editing, Writing – original draft, Data curation. **Huib A. Kerstjens:** Writing – review & editing, Writing – original draft, Data curation. **Alison Moore:** Writing – review & editing, Writing – original draft, Data curation. **David Slade:** Writing – review & editing, Writing – original draft, Data curation. **Robert A. Wise:** Writing – review & editing, Writing – original draft, Data curation. **Shiyuan Zhang:** Writing – review & editing, Writing – original draft, Software, Methodology, Conceptualization.

## Data availability

Data sharing is not applicable to this article as no datasets were generated or analyzed during the current study.

## Role of the funding source

This work was funded by GSK. The funders of the study had a role in the study design, data analysis, data interpretation and writing of the report.

## Declaration of competing interest

The authors declare the following financial interests/personal relationships which may be considered as potential competing interests.

**EP** was a GSK employee at the time of the study conception and holds financial equities in GSK. **DE, AM, DS** and **SZ** are GSK employees and hold financial equities in GSK. **GB** has received speaker fees from and served on advisory boards for AstraZeneca, Boehringer Ingelheim, Chiesi, GSK, Novartis and Sanofi. **PGG** has received speaker's fees and research grants from AstraZeneca, Chiesi, GSK, and Novartis. **HAK** has received research/educational grants and served on advisory boards for Boehringer Ingelheim, GSK and Novartis, and has served on advisory boards for AstraZeneca and Chiesi. **RAW** has received personal fees from AstraZeneca, Beyond Air, Boehringer Ingelheim, Contrafect, Roche-Genentech, Bristol Myers Squibb, Merck, Verona, Theravance, AbbVie, GSK, Chemerx, Kiniksa, Savara, Galderma, Kamada, Pulmonx, Kinevant, Vaxart, Polarean, Chiesi, 4D Pharma, Puretech, and grant support from AstraZeneca, Sanofi, Verona, Genentech, Boehringer Ingelheim, and 4DX imaging. He has received payment for expert testimony from the United States Government and Genentech; and support for attending meetings and/or travel from AstraZeneca. Additionally, he has received editorial support from GSK, AstraZeneca, Boehringer Ingelheim and Merck Foundation; and has served on the Board of Directors/Medical and Scientific Advisory Committee for the COPD Foundation, and on a Scientific Advisory Board for the American Lung Association.
